# Short-term outcome after open-heart surgery for severe chronic rheumatic heart disease in a low-income country, with comparison with an historical control group: an observational study

**DOI:** 10.1136/openhrt-2021-001706

**Published:** 2021-08-10

**Authors:** Ståle Wågen Hauge, Havard Dalen, Mette E Estensen, Robert Matongo Persson, Sintayehu Abebe, Desalew Mekonnen, Berhanu Nega, Atle Solholm, Marit Farstad, Nigussie Bogale, Torbjorn Graven, Niels-Erik Nielssen, Hege Kristin Brekke, Kjell Vikenes, Rune Haaverstad

**Affiliations:** 1 Department of Circulation and Medical Imaging, Norwegian University of Science and Technology, Trondheim, Norway; 2 Department of Heart Disease, Haukeland University Hospital, Bergen, Norway; 3 Clinic of Cardiology, St Olavs University Hospital, Trondheim, Norway; 4 Department of Medicine, Levanger Hospital, Levanger, Norway; 5 Department of Cardiology, Oslo University Hospital, Oslo, Norway; 6 Department of Clinical Science, Faculty of Medicine, University of Bergen, Bergen, Hordaland, Norway; 7 Department of Cardiology, School of Medicine, Addis Ababa University College of Health Sciences, Addis Ababa, Oromia, Ethiopia; 8 Department of Surgery, School of Medicine, Addis Ababa University College of Health Sciences, Addis Ababa, Oromia, Ethiopia; 9 Department of Surgical Services, Haukeland University Hospital, Bergen, Norway; 10 Clinic of Cardiology, Linköping University Hospital, Linköping, Sweden

**Keywords:** mitral valve stenosis, mitral valve insufficiency, global health, heart valve prosthesis implantation, outcome assessment, health care

## Abstract

**Objectives:**

Rheumatic heart disease (RHD) is a major burden in low-income and middle-income countries (LMICs). Cardiac surgery is the only curative treatment. Little is known about patients with severe chronic RHD operated in LMICs, and challenges regarding postoperative follow-up are an important issue. At Tikur Anbessa Specialised Hospital, Addis Ababa, Ethiopia, we aimed to evaluate the course and 12-month outcome of patients with severe chronic RHD who received open-heart surgery, as compared with the natural course of controls waiting for surgery and undergoing only medical treatment.

**Methods:**

Clinical data and outcome measures were registered in 46 patients operated during five missions from March 2016 to November 2019, and compared with the first-year course in a cohort of 49 controls from the same hospital’s waiting list for surgery. Adverse events were death or complications such as stroke, other thromboembolic events, bleeding, hospitalisation for heart failure and infectious endocarditis.

**Results:**

Survival at 12 months was 89% and survival free from complications was 80% in the surgical group. Despite undergoing open-heart surgery, with its inherent risks, outcome measures of the surgical group were non-inferior to the natural course of the control group in the first year after inclusion on the waiting list (p≥0.45). All except six surgical patients were in New York Heart Association class I after 12 months and 84% had resumed working.

**Conclusions:**

Cardiac surgery for severe chronic RHD is feasible in LMICs if the service is structured and planned. Rates of survival and survival free from complications were similar to those of controls at 12 months. Functional level and resumption of work were high in the surgical group. Whether the patients who underwent cardiac surgery will have better long-term prognosis, in line with what is known in high-income countries, needs to be evaluated in future studies.

**Key questions**

**What is already known about this subject?**

- Rheumatic heart disease (RHD) is a major cause of mortality and morbidity in low-income and middle-income countries (LMICs). In high-income countries, cardiac surgery is well established and recommended in severe RHD. There is limited availability of cardiac surgery and postoperative follow-up capacity in most LMICs, and knowledge about outcome after surgery in such settings is sparse.

**What does this study add?**

- Cardiac surgery and postoperative follow-up within an educational programme are feasible in an LMIC setting and the short-term outcome is acceptable.

**How might this impact on clinical practice?**

- Increased awareness of RHD as a major health problem in LMICs highlights the necessity of establishing programmes of cardiac surgery and postoperative follow-up. The involvement of skilled cardiac teams in establishing cardiac surgery programmes in LMICs may improve therapy for a large group of young individuals suffering from RHD and its complications.

## Introduction

Rheumatic heart disease (RHD) is globally a major cause of morbidity and mortality. Despite its near non-existence in the native population of the Western world, RHD is frequent in low-income and middle-income countries (LMICs). The Global Burden of Disease Study estimates that acute rheumatic fever (ARF) and subsequent (RHD) affect 33.4 million people worldwide, and it is estimated that the annual number of deaths due to RHD exceeds 300 000.^1^ Penicillin treatment of patients with RHD may reduce new episodes of ARF and thus postpone disease progression.^2 3^ Once severe RHD is present, medical therapy has no curative effect, leaving the patient with the option of surgery or inevitable death.^4–6^ Thus, establishing cardiac surgery for severe RHD in LIMCs has become an important issue.^3 7–11^ Even though RHD used to also be frequent in the Western world and cardiac surgery became the recommended treatment for advanced disease,^12–16^ the literature on such treatment in LMICs is scarce and includes limited follow-up data.^3 17–21^ For the same reasons, the natural course of severe RHD treated with modern medication is uncertain.^17 22–25^ In this study, we aimed to evaluate the perioperative course during open-heart surgery and at 12 months’ follow-up in patients with severe chronic valvular RHD, in an ongoing bilateral educational programme between Haukeland University Hospital, Norway, and Tikur Anbessa Specialised Hospital, Addis Ababa, Ethiopia. The primary purpose of our mission was to build a public cardiac surgery service in an LMIC institution with a planned framework that includes all necessary types of medical specialists in the heart team (box 1). Second, we aimed to provide high-quality open-heart surgery in chronic RHD patients and to maintain sustainability by establishing an educational programme with involvement of local medical professionals over a period of 10 years. Lastly, as presented in this study, we aimed to compare the surgical results with the course of a retrospective control group with similar characteristics and on medical therapy, who were on the same hospital’s waiting list of patients due to the limited cardiac surgery service. The main hypothesis was that a structured and planned open-heart surgery service for severe chronic RHD was feasible in this LMIC hospital and could provide acceptable short-term outcomes.

Box 1 **Overview of medical personnel provided for surgical service and education at black lion Hospital, Addis Ababa.**

**Types of medical professionals in the Norwegian heart team:**

Cardiac surgeon

Cardiologist (imaging/echocardiography, pacemaker and invasive cardiology)

Anaesthetist

Perfusionist

Anaesthesia nurse

Scrub nurse

Intensive care nurse

Ward nurse

Physiotherapist

Medical technician

## Methods

### Study design and population

**Figure 1:**
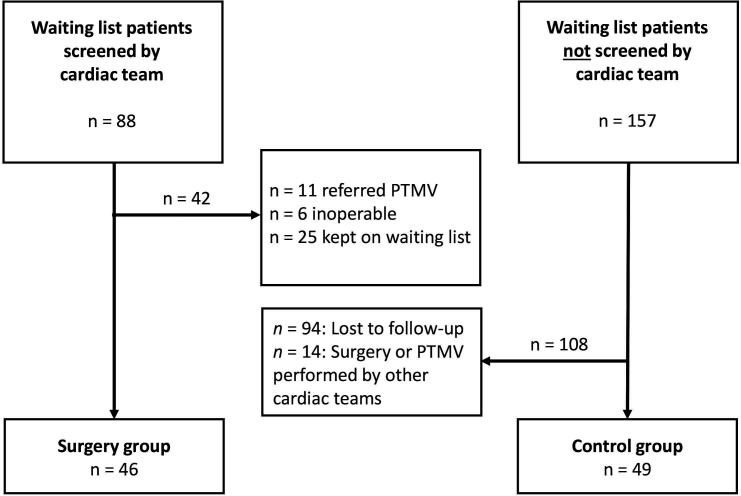
Flow chart of the study population. PTMV, percutaneous transluminal mitral valvuloplasty.

The study was observational in design, with detailed description of outcomes from the surgically treated patients and comparison with similar patients who were on medical therapy due to the absence of a cardiac surgery service. From the local RHD waiting list for open-heart surgery or percutaneous balloon valvuloplasty (PTMV), consisting of approximately 6000 patients, 88 were selected by the local staff for surgical screening by the Norwegian heart team during five missions from March 2016 to November 2019. Criteria for patient selection was long time on the waiting list for surgery, being considered eligible for intervention and compliant to follow-up. The surgical team had extensive experience in heart valve surgery of all kinds, including valve repair. The heart team chose the operative treatment, based on valve pathology, compliance with anticoagulation treatment and comorbidity, as well as family planning and rural housing issues. Of the screened 88 patients, 42 were excluded from or postponed for surgery, as shown in figure 1. Thus, 46 patients with RHD underwent cardiac surgery and were included in the surgical group for further analyses. No patient was included or excluded on the basis of socioeconomic background. From the same waiting list, 157 control patients on medical therapy were included based on similar characteristics for disease severity, age and sex at the time of placement on the waiting list. From this group, 14 underwent valvular surgery or PTMV by other missions or abroad and 94 were lost to follow-up; thus, the control group finally consisted of 49 patients (figure 1).

### Data collection

Data were collected by the heart team at screening and by Norwegian and local health professionals during follow-up. Anthropometric and blood pressure measurements were part of the clinical examinations. Body surface area (BSA) was calculated by the Mosteller formula. A 12-lead ECG was recorded for assessment of arrhythmias. Serum creatinine and other blood samples were collected during screening and analysed at the hospital laboratory. At follow-up, analyses were performed at Arsho Medical Laboratory, a certified private laboratory in Addis Ababa. International normalised ratio (INR) was measured using a hand-held CoaguCheck Pro II (Roche Diagnostics, Basel, Switzerland). The use of antibiotics, anticoagulation, diuretics, beta-blocker, ACE inhibitors and digoxin were registered. Heart failure symptoms were classified according to the New York Heart Association (NYHA) guidelines.^26^ At baseline, heart failure was defined by the following criteria: NYHA class ≥II, structural heart disease and at least one of the following: (1) at least two heart failure medications (furosemide, spironolactone, beta-blocker, ACE-inhibitor, digoxin); (2) left ventricular ejection fraction (LVEF) <50%; (3) peak tricuspid regurgitant velocity ≥3 m/s; (4) pleural effusion or ascites and (5) history of hospitalisation for heart failure. The EuroSCORE II calculator was used to assess risk for surgical patients.^27^

### Echocardiography

Echocardiography was performed by cardiologists from the Norwegian heart team and a local cardiologist in the surgical group and by local cardiologists only in the controls. In both groups, LVEF and volumes were calculated from apical four-chamber and two-chamber views (A4C and A2C) using the Simpson method. Tricuspid annular plane systolic excursion (TAPSE) was measured in right ventricular focused views by reconstructed motion mode. Similarly, systolic pulmonary artery pressure (SPAP) was calculated using the peak gradient across the tricuspid regurgitation using continuous Doppler (CW) in A4C, plus the right atrial pressure estimated from the size and respiratory variation of the inferior vena cava.^28^ LV stroke volume indexed for BSA and cardiac index was calculated using Doppler measurements. The severity of the rheumatic effect on the mitral valve was calculated using the Wilkins score.^29^ Grading of mitral stenosis (MS) was based on the valve area calculated using planimetry in parasternal short-axis views and/or by the pressure half-time of the mitral inflow Doppler spectrum (mitral valve area=220/pressure half-time).^30^ Grading of the severity of mitral regurgitation (MR) was based on valvular morphology, regurgitant jet flow colour and semiquantitative measurements such as vena contracta and signs of systolic pulmonary vein flow reversal. In addition, quantification of the regurgitant volume by proximal isovelocity hemispheric surface area was done in the surgical group. The grading of the severity of all valvular stenoses or regurgitations was done according to guidelines.^31 32^ All echocardiographic recordings were acquired using a GE Vivid E9 or a Vivid *i* ultrasound scanner (GE Ultrasound, Horten, Norway). All echocardiographic analyses of the surgical patients presented here were done offline using Echopac SWO, v. 201–203 (GE Ultrasound).

### Perioperative treatment, follow-up and outcome measures

Open-heart surgery with median sternotomy was performed using general anaesthesia and cardiopulmonary bypass circulation. All patients received perioperative evaluation by transoesophageal echo. All patients had a temporary pacemaker implanted during surgery; a permanent pacemaker (Medtronic) was later implanted if indicated by sustained postoperative conduction block. Patients with implanted mechanical valves or other indications for anticoagulation were treated with oral warfarin in accordance with international guidelines.^31^ Target INR for patients with mechanical aortic valve only, was 2.5 (range 2.0–3.0). For patients with a mechanical mitral valve (including those with more than one mechanical valve) target INR was 3.0 (range 2.5–3.5). Additional aspirin was not routinely used. Before postoperative discharge, all patients were evaluated with transthoracic echocardiography, laboratory testing and clinical evaluation. Regular follow-up was carried out at the hospital outpatient cardiac clinic. All patients were scheduled for evaluation after 6 and 12 months by a team consisting of local and Norwegian cardiologists and examined with comprehensive echocardiography, physical examination, 12-lead ECG, and laboratory testing. Optimised warfarin treatment was given special attention. For logistic reasons (ie, the ongoing pandemic), 16 patients did not get echocardiographic follow-up at exactly 12 months, but later on.

The perioperative outcome measures were mortality, need for permanent pacemaker, need for pericardial drainage, internal bleeding of any kind and thromboembolic events such as stroke or peripheral emboli. Events occurring within the first 30 days after surgery were classified as early postoperative complications. At 12 months, additional outcome measures were new onset of atrial fibrillation, infective endocarditis, valvular thrombosis and heart failure hospitalisation. The first occurrence of any postoperative event was used for outcomes in the surgical group. Similarly, the first occurrence of any event after inclusion on the waiting list was used in the control group. NYHA class and resumption of professional activity for surgical patients were also registered. Outcome measures of the control group were collected by searches of electronic and printed medical reports, as well as outpatients’ visits and telephone calls.

End points were defined as follows: Atrial fibrillation, as diagnosed by ECG. Stroke was defined as physician-confirmed sudden onset of neurological deficit consistent with ischaemia of a cerebral vascular territory, lasting ≥24 hours, with or without neuroimaging evidence. Peripheral embolic episode was diagnosed clinically by loss of arterial pulse and clinical signs of end-organ ischaemia (ie, ischaemic limb pain or gangrene). If cardiac or prosthetic heart valve thrombosis was suspected by transthoracic echocardiography, supplementary transoesophageal examination and/or fluoroscopy was used to confirm the diagnosis. Bleeding event was defined as symptoms and/or findings consistent with internal bleeding, without specified drop in haemoglobin. Infective endocarditis was defined according to the modified Duke criteria.^33^ Heart failure admissions were defined by hospital readmission with clinical diagnosis of heart failure, even if natriuretic peptides or echocardiograms were not acquired. Resumption of professional activity was defined by a 100% return to work or if the patient had resumed their studies.

### Statistical analyses

Continuous variables are expressed as mean and SD or as median and IQR, as appropriate. Normality was evaluated using histograms and normality plots. Categorical variables are presented as frequencies and proportions. The Student’s t-test and Wilcoxon test were used for comparisons of groups or sexes when appropriate. Proportions were compared using the χ^2^ test and Fisher’s exact test. Survival analyses were performed using the Kaplan-Meier method. The starting point for inclusion in the study was defined as the date of open-heart surgery for the surgical group and the date of inclusion on the open-heart surgery waiting list for controls. No missing data were imputed.

In case no event had occurred, patients and controls were censored at 12 months from surgery or inclusion on the waiting list, respectively. Values of p<0.05 were considered statistically significant. All statistical analyses were performed using IBM SPSS Statistics V.26.

## Results

### Population and surgery

The baseline characteristics of the 46 surgical patients and 49 controls are shown in table 1. Both groups had characteristics of symptomatic severe RHD. The controls were approximately 4 years younger, with a mean age of 26 years as compared with 30 years in the surgical group (p=0.04). Females were overrepresented in both groups (65% of the surgical group and 76% of the controls). In the surgical group, height, weight, and BSA differed between sexes, but BMI was similar. The surgical patients had been symptomatic for a median of 7 years longer before inclusion in the study (time of surgery) compared with controls (enrolled at the time of inclusion on the waiting list) (p<0.01). The surgical group had more advanced disease than the control group, with higher proportions of patients with atrial fibrillation (p<0.01), use of anticoagulation (p<0.01) and use of beta-blocker (p<0.01), respectively. Furthermore, more patients in the surgery group had experienced thromboembolic events compared with the controls. EuroSCORE II was higher for women than for men, at 3.3% and 1.6%, respectively.

**Table 1:** Basic characteristics of the study population

Variables	Surgical group (n=46)	Control group (n=49)	P value surgical versus controls
Female, n (%)	30 (65)	37 (76)	0.27
Age, mean (SD), years*	30.0 (8.6)	25.9 (9.9)	0.04
NYHA class III–IV, n/a (%).	21/46 (46)	21/37 (57)	0.30
Height, mean (SD), cm*	162 (9.5)	–	
Weight, mean (SD) kg*	57.3 (13.1)	–	
Body mass index, mean (SD), kg/m^2^†	21.8 (4.3)	–	
Body surface area, mean (SD), m† *	1.59 (0.20)	–	
Blood pressure systolic, mean (SD), mm Hg	117 (21)	107 (15)	<0.05
Blood pressure diastolic, mean (SD), mm Hg	73 (11)	66 (12)	<0.05
Heart rate, median (IQR), per minute	80 (74–90)	80 (78–92)	0.17
Serum creatinine, mean (SD) mg/dL	0.93 (0.25)	–	
Time on waiting list/symptoms, median (IQR), years†	10 (6–15)	3 (1–5)	<0.01
Antibiotics n/a (%)	40/46 (87)	–	
Anticoagulation, n/a (%)	26/46 (57)	6/42 (14)	<0.01
Beta-blocker, n/a (%)	19/46 (41)	6/49 (12)	<0.01
Diuretics, n/a (%)	35/46 (76)	34/48 (71)	0.56
Left ventricular ejection fraction ≤50, n/a (%)	10/43 (23)	3/36 (8)	0.08
Peak systolic artery pressure ≥60, n/a (%), mm Hg	20/43 (47)	10/15 (67)	0.18
Numbers of valves affected, mean (SD)	1.8 (0.7)	1.7 (0.7)	0.66
EuroSCORE II (%)*	2.7 (1.7)	–	
Complications of RHD:			
Atrial fibrillation, n/a (%)	23/46 (50)	7/39 (18)	<0.01
Thromboembolic disease, n/a (%)‡	6/46 (13)	0/41 (0)	0.02
Stroke, n/a (%)	2/46 (4)	0/41 (0)	0.18
Infective endocarditis, n/a (%)*	2/46 (4)	2/41 (5)	0.91
Heart failure, n/a (%)§	36/46 (78)	36/49 (74)	0.59

*Sex differences in the surgery group: age, women 28.1 versus men 33.6 years; height, women 156.7 vs men 171.4 cm; weight, women 53.5 vs men 64.3 kg; BSA, women 1.52 vs men 1.75 m^2^; infective endocarditis, women 0% versus men 13%; EuroSCORE II, women 3.3 vs men 1.6.

†For surgical patients: years of symptoms before surgery; for controls: years of symptoms before inclusion on the waiting list.

‡Thromboembolic disease: peripheral emboli, left atrial thrombus and valve thrombus.

§Heart failure: NYHA ≥2, structural heart disease and at least one of the following: >2 heart failure medicines, EF <50, TR v/max >3 m/s, pleural effusion/ascites, admissions with heart failure.

BSA, body surface area; EF, ejection fraction; n, numbers; n/a, numbers/available; NYHA, New York Heart Association; TR, tricuspid regurgitant.

### Echocardiography

Detailed echocardiographic data for the surgical group are summarised in table 2. Mitral valve disease was the most common valve defect, with severe MS (≤1.5 cm^2^) in 62% and critical MS (≤1.0 cm^2^) in 35%. Furthermore, 62% had moderate or severe MR, and 28% had combined MS and MR. The average Wilkins score was high (11). LVEF was within normal range (mean 56%), whereas median cardiac index and indexed LV stroke volume were low (2.2 L/min/m^2^ and 28 mL/m^2^, respectively). SPAP was elevated, with a mean of 56 mm Hg.

**Table 2:** Echocardiographic measurements from the surgical group

Variable	N available	Mean (95% CI)
Left ventricular dimension, end-diastolic, mm	45	53 (50 to 56)
Left ventricular ejection fraction, %	46	56 (53 to 58)
Left ventricular end-diastolic volume, mL	38	126 (106 to 147)
Indexed left ventricular stroke volume, median (IQR), mL/m^2^.	34	28 (22 to 43)
Cardiac index, median (IQR), L/min/m^2^	34	2.2 (1.8 to 4.2)
Indexed left atrial volume, median (IQR), mL/m^2^	38	102 (71 to 186)
Tricuspid annular plane systolic excursion, mm	44	20 (18 to 21)
Systolic pulmonary arterial pressure, mm Hg	41	56 (50 to 62)
Severe mitral stenosis*, n (%)	46	28 (62)
Very severe mitral stenosis†, n (%)	46	16 (35)
Mean gradient mitral valve‡, mm Hg	28	13 (11 to 15)
Mitral valve area§, cm^2^	27	0.99 (0.87 to 1.12)
Moderate to severe mitral regurgitation, n (%)	46	28 (61)
PISA mitral regurgitation volume¶, mL	14	71 (54 to 88)
Wilkins score**	37	11 (10 to 12)
Moderate to severe aortic stenosis and/or aortic regurgitation, n (%)	46	14 (30)
Moderate to severe tricuspid stenosis and/or tricuspid regurgitation, n (%)	46	25 (54)

Values are mean (95% CI) if not otherwise specified.

*Mitral valve area <1.5 cm^2^.

†Mitral valve area <1.0 cm^2^.

‡Mean gradient given for patients with severe mitral stenosis.

§Mitral valve area given for patients with severe mitral stenosis.

¶Regurgitant volume by PISA, given for patients with mod-severe mitral regurgitation.

**Wilkins score given for patients with rheumatic mitral disease.

n, numbers; PISA, proximal isovelocity surface area.

Details of concomitant surgical procedures are shown in table 3. Most patients needed multiple heart surgery procedures, with means of 1.8 valvular and 2.4 total procedures performed per patient. The mitral valve was the most commonly diseased valve (93%). Mechanical valve implantation was the most common valvular procedure. Similarly, tricuspid valve repair was the most common valvular reconstruction accounting for more than half of the total, whereas mitral and aortic valve repair were performed in 7 (15%) and 2 (4%), respectively. Surgical repair procedures included annuloplasty, commissurotomy, leaflet extension and subvalvular procedures.

**Table 3:** Surgical data and complications during the 30-day postoperative period

Specification	Total (n=46)	Women (n=30)	Men (n=16)	P value sex difference
Extracorporeal circulation time, mean (SD), min	151 (46)	148 (41)	157 (56)	0.57
Aortic cross clamp time, mean (SD), min	111 (39)	109 (38)	117 (43)	0.48
Procedures per patient, mean (SD)*	2.4 (1.0)	2.6 (0.8)	2.1 (1.3)	0.15
Valve procedures per patient, mean (SD)	1.8 (0.7)	1.9 (0.6)	1.6 (0.7)	0.10
Aortic valve procedures, n	14	7	7	
Replacement; mechanical prosthesis, n (%)	12 (26)	5 (17)	7 (44)	<0.05
Aortic valve repair, n (%)	2 (4)	2 (7)	0 (0)	0.29
Mitral valve procedures, n	43	30	13	
Replacement; mechanical prosthesis, n (%)	35 (76)	24 (80)	11 (67)	0.33
Replacement; biological prosthesis, n (%)	1 (2)	0 (0)	1 (6)	0.33
Mitral valve repair, n total (%), (ring- commissurotomy-leaflet extension-subvalvular†)	7 (15), (7-3-2-7)	6 (20), (6-3-1-6)	1 (6), (1-0-1-1)	0.21
Tricuspid valve procedures, n	25	20	5	
Replacement; biological prosthesis, n (%)	1 (2)	0 (0)	1 (6)	0.16
Tricuspid valve repair, n (%) (ring; others‡)	24 (52), (21; 3)	20 (67), (17; 3)	4 (25), (4; 0)	<0.05
Complications perioperatively and postoperatively				
Mortality <30 days, n (%)	3 (7)	3 (10)	0 (0)	0.19
Permanent pacemaker implantation, n (%)	5 (11)	4 (13)	1 (6)	0.46
Pericardial drainage, n (%)	1 (2)	1 (3)	0 (0)	0.46
Stroke, n (%)	2 (4)	1 (3)	1 (6)	0.64

*Including valvular procedures and others such as left atrial appendage closure, left atrial thrombectomy, atrial septal defect closure secondary to failed balloon angioplasty, etc.

†Subvalvular procedures (one with debridement of subvalvular abscess; the others all include transition of chordae, placement of neochordae and cleavage of subvalvular apparatus).

‡Ring plasty, commissurotomy and subvalvular procedures.

### Early outcomes

Three patients died within the first 30 days, including one due to severe pulmonary hypertension refractory to medication. Another patient who initially was well mobilised died following in-hospital cardiac arrest. Low serum potassium level was confirmed in this patient. The third early death occurred after re-admittance for septicaemia of unknown origin and accompanying diarrhoea 23 days after surgery.

Five (11%) patients, all of whom had multi-valvular surgery, including four with tricuspid valve repair, developed complete atrioventricular conduction block after surgery and received permanent pacemaker implantation. Of the latter, two had atrial fibrillation prior to surgery. Furthermore, one patient (2%) needed percutaneous pericardial drainage early postoperatively. Two patients (4%) suffering from perioperative stroke recovered with minimal sequelae.

### One-year outcomes

Five (11%) of the surgical patients died within the first 12 months. Of the subsequent deaths, one patient died suddenly at home 64 days after surgery without any prior symptoms; ventricular arrhythmia was therefore suspected. The last death was a sudden cardiac arrest 10 months after surgery. The female patient was first admitted with excessive pericardial effusion due to post-pericardiotomy syndrome, which was successfully treated with a pericardial window to the left chest at 6 months follow-up, and she was discharged in good shape. She died suddenly 4 months later, probably due to arrhythmia, as she was given a combination of digoxin and diuretics with poor local medical follow-up.

Additional outcomes at 12 months postsurgery were admissions for heart failure in two patients. No surgical patient developed prosthetic valve dysfunction or suffered from infectious endocarditis, thromboembolism or major bleeding during the follow-up. Two surgical patients (6%) had new onset of atrial fibrillation, whereas two patients with atrial fibrillation at baseline had regained sinus rhythm at the 12 month follow-up.

**Figure 2:**
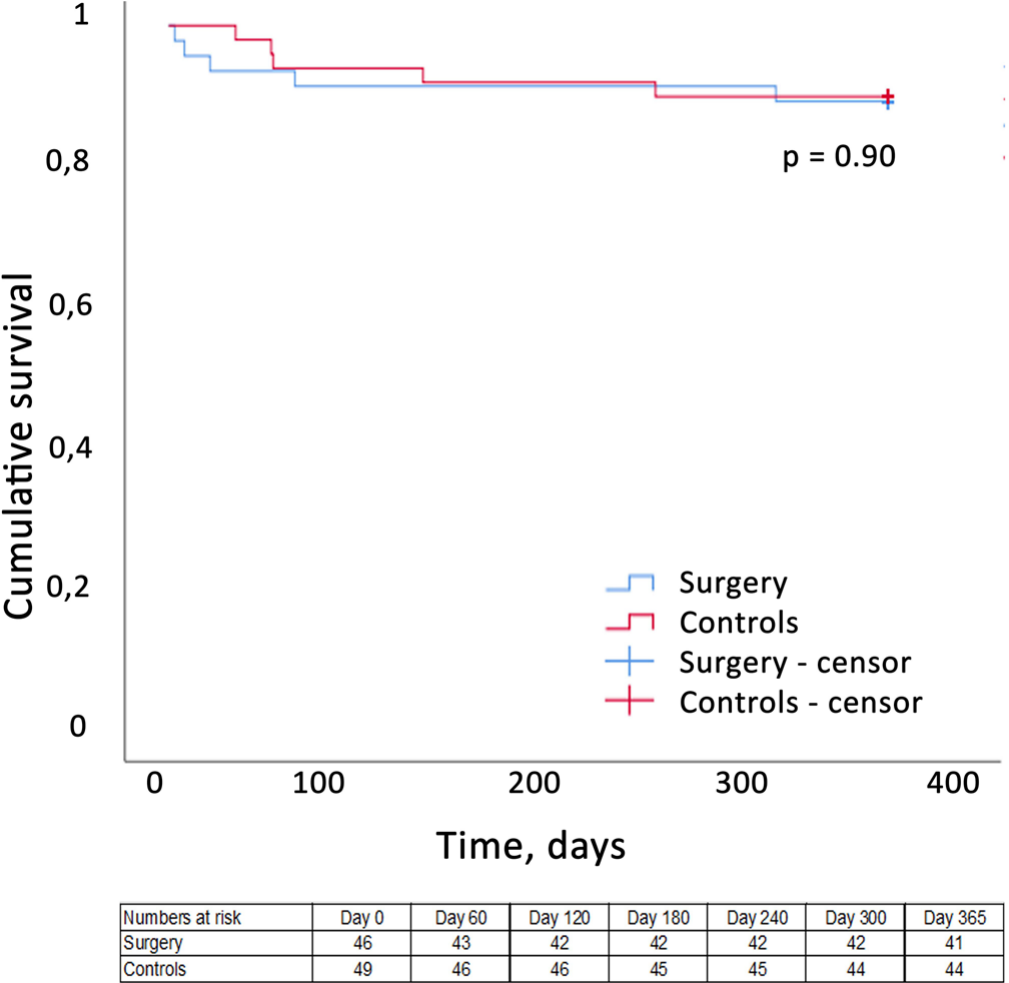
Survival at 12-month follow-up. Surgical group (blue) and the control group (red). Participants without events are censored at 365 days.

**Figure 3:**
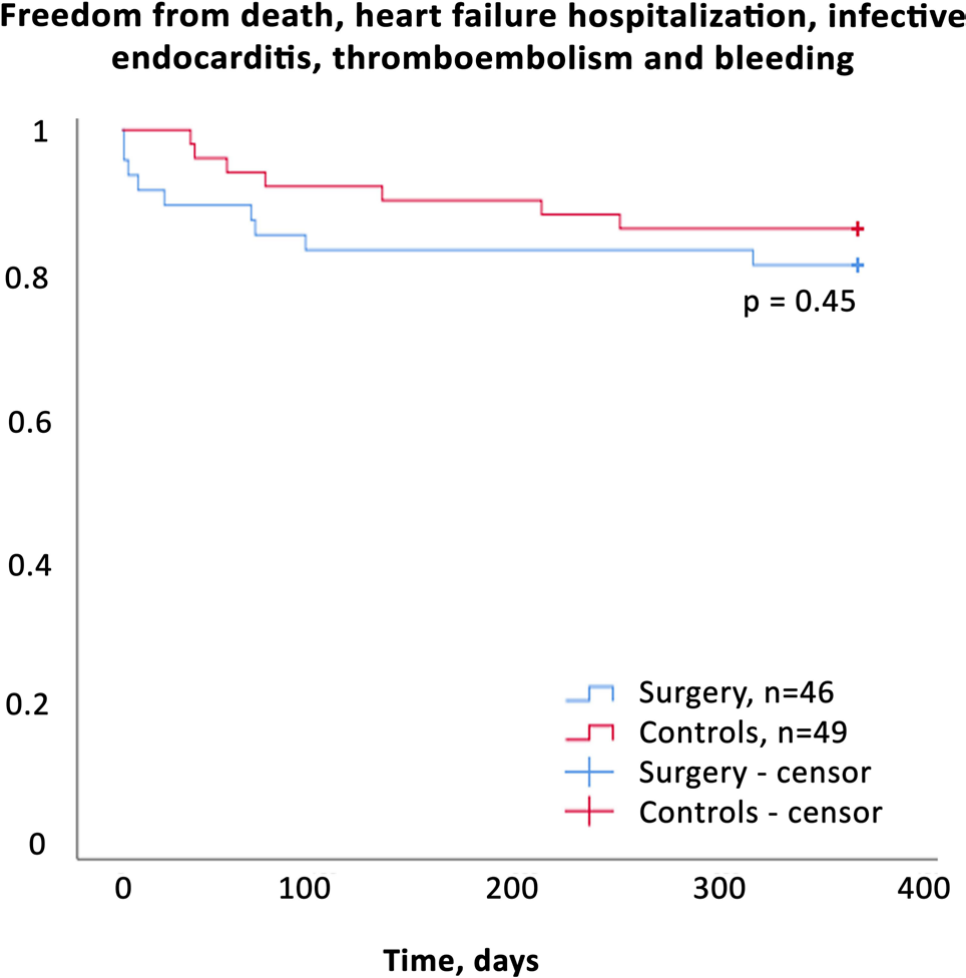
Survival free from complications at 12-month follow-up. The surgical group (blue) and the cohort of historical controls (red). All participants without events are censored at 365 days, and no patient was lost to follow-up.

Five control patients (10%) died during the first 12 months of follow-up and two were readmitted with heart failure. The survival rates at 12 months were 89% in the surgical group and 90% in the controls, with no difference between groups (figure 2). Survival free of complications was 80% in the surgical group and 86% in the controls, with no significant difference (figure 3). Importantly, no surgical or control patient was lost to follow-up. All patients except six (85%) were in NYHA class I at 12 months following surgery, and 84% of the patients had returned to their professional activity. Tables 3 and 4 show the favourable outcomes at the 12-month follow-up postsurgery.

**Table 4:** Clinical outcome at 12-month follow-up

Variables	Surgical group total (n=46)	Control group total (n=49)	P value surgical group versus control group
NYHA 1 at 12 months, n (%)	35 (85)	–	
Back to professional activity, n/a (%)*	27/32 (84)	–	
**Total complications 12 months**			
Mortality, n (%)	5 (11)	5 (10)	0.90
Stroke, n (%)	2 (5)	0 (0)	0.13
Heart failure hospitalisation, n (%)	2 (5)	2 (5)	0.94
New onset AFIB, n/a (%)*	2/32 (6)	–	

No patients experienced IE, other thromboembolic events or bleedings.

*Missing data on nine patients in the surgical group.

AFIB, atrial fibrillation; NYHA, New York heart association functional classification; n/a, numbers/available.

## Discussion

The study presents current information on short-term outcome following open-heart surgery for severe chronic RHD and the natural history of conservatively treated RHD in LMICs. Despite a number of challenging medical and logistic factors, cardiac surgery for severe long-standing RHD is feasible in such environments if the programme is well structured and planned. Furthermore, all surgery and follow-up were part of an educational programme that aims to create a sustainable framework for a recently established cardiac service. Both survival and survival free of complications at 12 months were equal to those of patients with similar characteristics getting medication only. Important for comparison of the two groups is the more advanced clinical status of the operated group, as the surgical patients were included after a median 10 years on the waiting list, while the natural course of the controls reflects the first year after referral. If proper follow-up is maintained, it is expected that patients who have been successfully operated will do better in the long term as compared with patients receiving conservative treatment.^5 6^

### Population

As a randomised study and propensity matching would be unrealistic, the characteristics of the two groups were balanced with respect to sex and severity of valvular disease at the time of inclusion on the waiting list. However, the control group was younger, as follow-up started at inclusion on the waiting list compared with the date of surgery in the intervention group. As 94 (60%) of the eligible controls were excluded due to missing data, the distribution of sex and disease severity differed somewhat between the surgical patients and the controls, with more female patients in the control group and more severe disease and comorbidities in the surgical patients. Thus, the controls reflect the natural course of the disease itself.

The medical treatment differed between the groups. Whether the observations of less atrial fibrillation, less use of anticoagulation and fewer thromboembolic events among the controls are related to younger age and shorter time since referral or less comprehensive assessment is uncertain. In line with previous studies, we present a population of predominantly females of young age with severe rheumatic valvular disease. Compared with a recent publication, this population had more long-standing and severe symptoms, and congestive heart failure was more common.^4^ For surgical patients, SPAP was significantly elevated (mean 56 mm Hg) and 24 (52%) had reduced RV function (TAPSE <20 mm). Even though LVEF was >50% in 33 (77%) of the patients, median indexed stroke volume was only 28 mL/m^2^, which indicates poor LV function in a large proportion of the patients. Together with the young age of the population, this indicates that the relatively low EuroSCORE II in the population probably does not truly reflect the surgical risk, an opinion in accord with previous reports.^34^

### Outcomes

Follow-up data after cardiac surgery for severe chronic RHD in LMICs is sparse. The observational design and inherent differences between the groups indicate that comparisons between the groups should be interpreted with caution. The mortality rate and survival free from complications within 12 months after surgery were comparable to the natural course of symptomatic RHD patients with similar characteristics. However, there were great differences in the grade of RHD and symptom duration between the groups, which were in favour of the control group within 12 months’ follow-up. The early postoperative deaths may be attributed to the setting in which local experience and resources were scarce. Some of these deaths would probably be avoided in societies with optimal medical follow-up programmes and enough resources.

Our survival rates after cardiac surgery in LMICs are in line with others, but in numerous previous reports a large proportion of the patients were lost to follow-up, even during the first year, and the mortality rates might have been underestimated as a result.^18 20 21 35^ Thus, a strength of this study is the complete follow-up of all surgical patients. The outcome for the control group is also in line with those of previous publications.^4 24^ However, a substantial proportion of patients eligible as controls were excluded after further analysis, as data were missing that could not be updated even after several attempts. Thus, we also believe that studies of control groups of patients with severe chronic RHD on medical therapy generally underestimate mortality and severe complications.

Among the main concerns following implantation of mechanical valves were complications related to anticoagulation therapy and pregnancy. We did not see any complications from anticoagulation or pregnancy during 12 months’ follow-up, although comprehensive information and scrutiny were given to reveal bleeding complications and prosthetic valve dysfunction. Female patients were advised not to become pregnant in the first year after surgery. The establishment of an INR clinic especially for the surgical patients, organised by a local pharmacist and supported by local physicians, played an important role in proper INR monitoring and it enhanced patient awareness about this critical issue.

The present study was encouraging concerning the clinical effects of cardiac surgery on cardiac symptoms and functional improvement in RHD patients. Importantly, surgical patients experienced far fewer symptoms postoperatively, in line with previous findings,^35^ and most resumed professional activity.

### Ethical considerations

A randomised controlled study would not be ethically acceptable. As cardiac surgery service was unavailable to a large group of patients in need of surgery, we were able to construct a control group that added information regarding the natural history of advanced RHD in the LMIC setting. Partly based on ethical considerations, patients with the most severe disease but still assessed as operable by the heart team were prioritised for surgery. In some cases, the patients were technically operable, but declined surgery as they carried too high a surgical risk due to severe heart failure, critical pulmonary hypertension or severe sequelae following previous stroke from cerebral embolisation. These categories of patients would have to undergo preoperative optimised medical treatment or would require robust intensive care facilities and postoperative rehabilitation programmes currently unavailable in LMICs. Patients screened by the heart team but not accepted for operation were not included as controls.

### Limitations

The main limitation is the low number of participants and controls. However, none of the included patients was lost to follow-up, which is uncommon in studies originating from LMICs. The design is observational, and comparisons between the surgical and control group should be interpreted with caution as they are not equal groups. The surgical patients were symptomatic for a longer time, even though they had similar characteristics at the time of their inclusion on the waiting list. We lack comprehensive data on comorbidity and some outcome data in the control group. Despite this, our findings add to existing knowledge concerning outcomes for surgical RHD patients in LMICs. The global coronavirus pandemic has influenced the number of surgeries and their follow-up and may have also worsened the outcome in some of the patients, as this most unfortunate event is an extraburden on healthcare systems, especially in LMICs.

## Conclusion

Outcome within 12 months after open-heart surgery for severe chronic RHD in a sub-Saharan country was acceptable as compared with the natural history of RHD in a control group with shorter disease duration. Most patients improved to NYHA class I and resumed professional activity. With a proper selection of patients and a structured team approach, surgery with acceptable short-term results for this group of patients is achievable. Careful cardiac follow-up and adequate anticoagulation is mandatory. Evaluation of long-term outcomes is important for future treatment of this large group of young individuals with severe RHD in LMICs.

## Data Availability

Data are available on reasonable request to the authors.
